# Predictive power of deep-learning segmentation based prognostication model in non-small cell lung cancer

**DOI:** 10.3389/fonc.2023.868471

**Published:** 2023-04-04

**Authors:** Jordan C. Gainey, Yusen He, Robert Zhu, Stephen S. Baek, Xiaodong Wu, John M. Buatti, Bryan G. Allen, Brian J. Smith, Yusung Kim

**Affiliations:** ^1^ Department of Radiation Oncology, The University of Iowa, Iowa City, IA, United States; ^2^ Department of Data Science, Grinnell College, Grinnell, IA, United States; ^3^ Department of Data Science, University of Virginia, Charlottesville, VA, United States; ^4^ Department of Radiation Oncology, MD Anderson Cancer Center, Houston, TX, United States

**Keywords:** prognostication, non-small cell lung cancer, deep learning, RECIST (response evaluation criteria in solid tumors), lung cancer

## Abstract

**Purpose:**

The study aims to create a model to predict survival outcomes for non-small cell lung cancer (NSCLC) after treatment with stereotactic body radiotherapy (SBRT) using deep-learning segmentation based prognostication (DESEP).

**Methods:**

The DESEP model was trained using imaging from 108 patients with NSCLC with various clinical stages and treatment histories. The model generated predictions based on unsupervised features learned by a deep-segmentation network from computed tomography imaging to categorize patients into high and low risk groups for overall survival (DESEP-predicted-OS), disease specific survival (DESEP-predicted-DSS), and local progression free survival (DESEP-predicted-LPFS). Serial assessments were also performed using auto-segmentation based volumetric RECISTv1.1 and computer-based unidimensional RECISTv1.1 patients was performed.

**Results:**

There was a concordance between the DESEP-predicted-LPFS risk category and manually calculated RECISTv1.1 (φ=0.544, p=0.001). Neither the auto-segmentation based volumetric RECISTv1.1 nor the computer-based unidimensional RECISTv1.1 correlated with manual RECISTv1.1 (p=0.081 and p=0.144, respectively). While manual RECISTv1.1 correlated with LPFS (HR=6.97,3.51-13.85, c=0.70, p<0.001), it could not provide insight regarding DSS (p=0.942) or OS (p=0.662). In contrast, the DESEP-predicted methods were predictive of LPFS (HR=3.58, 1.66-7.18, c=0.60, p<0.001), OS (HR=6.31, 3.65-10.93, c=0.71, p<0.001) and DSS (HR=9.25, 4.50-19.02, c=0.69, p<0.001). The promising results of the DESEP model were reproduced for the independent, external datasets of Stanford University, classifying survival and ‘dead’ group in their Kaplan-Meyer curves (p = 0.019).

**Conclusion:**

Deep-learning segmentation based prognostication can predict LPFS as well as OS, and DSS after SBRT for NSCLC. It can be used in conjunction with current standard of care, manual RECISTv1.1 to provide additional insights regarding DSS and OS in NSCLC patients receiving SBRT.

**Summary:**

While current standard of care, manual RECISTv1.1 correlated with local progression free survival (LPFS) (HR=6.97,3.51-13.85, c=0.70, p<0.001), it could not provide insight regarding disease specific survival (DSS) (p=0.942) or overall survival (OS) (p=0.662). In contrast, the deep-learning segmentation based prognostication (DESEP)-predicted methods were predictive of LPFS (HR=3.58, 1.66-7.18, c=0.60, p<0.001), OS (HR=6.31, 3.65-10.93, c=0.71, p<0.001) and DSS (HR=9.25, 4.50-19.02, c=0.69, p<0.001). DESEP can be used in conjunction with current standard of care, manual RECISTv1.1 to provide additional insights regarding DSS and OS in NSCLC patients.

## 1 Introduction

Lung cancer is the leading cause of cancer-related death worldwide accounting for 1.8 million deaths per year (1). According to the American Cancer Society, the five-year survival rate of lung cancer is 19% in the United States (2). Stereotactic body radiotherapy (SBRT) is an established treatment option for patients with early stage non-small cell lung cancer (NSCLC) with over 90% local control and 56% survival at three-years (3). Utilization of SBRT continues to increase in recent years (4). Given the localized nature of SBRT treatment, patients remain at risk for disease recurrence in the untreated regions of the lung with five-year intra-thoracic recurrence rates of 20% (5). Surveillance imaging with an accurate method of identifying progressive disease is vital to identify recurrence and maintain cancer control.

The Response Evaluation Criteria in Solid Tumors (RECISTv1.0) was introduced in 2000 to systematically categorize target lesions on cross-sectional imaging (6). These guidelines were updated to version 1.1 (RECISTv1.1) in 2008 with clarifications published in 2016 (7, 8). RECISTv1.1 utilizes linear tumor measurements for categorizing response to treatment. Given the often irregular spiculated appearance of NSCLC, there is significant intraobserver and interobserver variability in the measurement of lesions on imaging (9). Attempts have been made to expand upon RECISTv1.1 including a set of guidelines which utilizes positron emission tomography (PET) imaging to evaluate functional changes within a tumor (10). Volumetric measurements from computed tomography (CT) images have also been studied and may have a higher correlation with overall survival (11). Estimating tumor volume using an ellipsoid model may correlate with overall survival and utilizes the same numerical thresholds as RECISTv1.1 (12).

Deep learning algorithms have attracted a tremendous amount of attention in the field of medical imaging and can provide advanced quantitative analysis in medical imaging data. Previous hypothesis-generating work suggested that features captured by a convolutional neural network (CNN) trained for the purpose of automatic tumor segmentation can identify radiomic characteristics which are highly correlated with survival despite that the features themselves had never been supervised with any survival-related information (13). Deep learning algorithm studies have been reviewed in prognostics and health management (14) and different AI architectures of prediction models (15). The deep learning application in lung cancer prognostications has been widely investigated using patient histology (16), integrating biological microarray information with clinical data (17), genomic information (18), CT images (19), and PET-CT images (13). Deep learning prognostication performance has been investigated for lung cancer patients who received surgery (20), radiotherapy (13), and immunotherapy (21). However, the prognostication performance of deep-learning based algorithms in local progression free survival (LPFS), disease specific survival (DSS), and overall survival (OS) has not been fully and quantitatively compared with those of RECISTv1.1. The current study seeks to expand upon this previous work (13) and employ a similar deep learning segmentation based prognostication (DESEP) strategy which could be used in conjunction with RECISTv1.1 to predict local progression free survival (LPFS), disease specific survival (DSS), and overall survival (OS). The DESEP model utilized solely pre-treatment CT images for prediction.

In this study, the prognostication performance of RECISTv1.1 in LPFS was assessed in comparison with the DESEP model. In addition, the limitations of RECISTv1.1 in OS and DSS prediction was discovered in contrast to the promising predictive performance of the DESEP model.

## 2 Materials and methods

### 2.1 Patient characteristics

A total of 108 subjects were analyzed retrospectively following approval from the Institutional Review Board (IRB: 200503706). Patient demographics and clinical characteristics were summarized in **Table 1**. All patients provided consent for the use of their clinical information and medical images and signed an informed consent form approved by the Institutional Review Board. All data collection and experimental procedures are in accordance with relevant guidelines and regulations. All patients underwent SBRT for NSCLC with treatments ranging from July 2006 to October 2018. The SBRT plans were generated using intensity-modulated radiotherapy (IMRT) in a form of either step-and-shoot in Oncor (Siemens Medical Solutions USA, Inc., Malvern, PA, USA), or volumetric-modulated radiotherapy (VMAT) in VersaHD (Elekta Inc., Atlanta, GA, USA). The SBRT patients received 12Gy/fraction in 4 fractions (12 X 4), 10 X 5, or 16~18 X 3 with daily cone-beam CT guidance and surface monitoring (VisionRT, London, UK). Target volumes were delineated by radiation oncologists using both CT and PET imaging, and contouring was completed using Velocity AI (Varian Medical System, Inc., Palo Alto, CA). Following SBRT, patients were followed with surveillance CT images at approximately 2 months following SBRT then every 3 months thereafter.

**Table 1:** Patient demographics and clinical characteristics.

Patient Population		Prior Treatment	
Age at SBRT	72.0 years (± 9.7 years)	Previous Radiation Therapy	33 (30.6%)
Mean Overall Survival	2.0 years (± 1.4 years)	Previous Surgery	31 (28.7%)
Mean Disease Specific Survival	1.8 years ( ± 1.4 years)	Previous Chemotherapy	41 (38.0%)
Mean Local Progression Free Survival	1.6 years (± 1.3 years)	Previous Immunotherapy	2 (1.9%)
Male	51 (47.2%)	**Stage**	
Female	57 (52.8%)	IA	51 (47.2%)
**Survival**		IB	16 (14.8%)
Alive	50 (46.3%)	IIA	3 (2.8%)
Dead	58 (53.7%)	IIB	3 (2.8%)
**Cause of Death**		IIIA	5 (4.6%)
Alive	50 (46.3%)	IIIB	16 (14.8%)
Cancer-related	40 (37.0%)	IV	14 (13.0%)
Not Cancer-related	11 (10.2%)	**Karnofsky Performance Score**	
Unknown	7 (6.5%)	100	5 (4.6%)
**Local Progression Free Survival**		90	25 (23.1%)
Survived without Progression	36 (33.3%)	80	40 (37.0%)
Progression or Death	72 (66.7%)	70	30 (27.8%)
**2 Year Overall Survival**		60	6 (5.6%)
Survived	50 (46.3%)	<60	2 (1.9%)
Died	42 (38.9%)	**Histology**	
Insufficient Follow-up	16 (14.8%)	Adenocarcinoma	55 (50.9%)
**2 Year Disease Specific Survival**		Squamous Cell Carcinoma	41 (38.0%)
Survived	57 (52.8%)	Adenosquamous	2 (1.9%)
Died	35 (32.4%)	Metastasis from Prior NSCLC	1 (0.9%)
Insufficient Follow-up	16 (14.8%)	Clinical Diagnosis	9 (8.3%)
**2 Year Local Progression Free Survival**			
Survived	28 (25.9%)		
Died/Progressed	64 (59.3%)		
Insufficient Follow-up	16 (14.8%)		

Continuous data are presented in the form mean ( ± standard deviation), discrete data are presented in the form number (percentage).

There were a total of 51 male and 57 female patients represented in this study. There were 55 patients with adenocarcinoma, 41 with squamous cell carcinoma, 12 adenosquamous, 1 with metastasis from previous NSCLC, and 9 without a biopsy. The patients’ prognostic stage varied and included 67 patients with stage I, 6 patients with stage II, 21 patients with stage III, and 14 patients with stage IV disease. The patients’ stage were classified by the eight edition American Joint Committee on Cancer (AJCC) staging manual (22). By the end of the study, 72 patients had experienced local progression, 58 patients had died, and 40 of those deaths were cancer-related.

### 2.2 Deep-learning segmentation based prognostication (DESEP) model

A 3D segmentation algorithm was developed using a U-Net architecture.^14^ This architecture has an “hourglass” structure which extracts imaging features at varying levels of granularity. The input of the CT Segmentation U-Net is a cropped 3D CT image measuring 96 x 96 x 48 mm^3^ with the tumor located in the center of the image. The target output of the U-Net is a segmentation mask trained on the ground truth of our study which is a binary mask map defined by three radiation oncologists’ contours of the gross tumor volume aggregated by the STAPLE algorithm.^15^ Through training the segmentation U-Net, we have achieved over 75% of segmentation accuracy measured by dice similarity coefficient.

**Figure 1:**
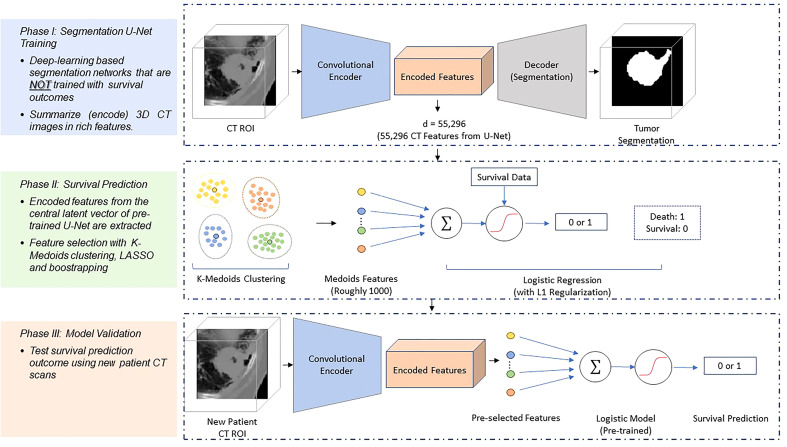
Schematic diagram of the survival prediction architecture. The DESEP model consists of two major phases: the U-Net segmentation (Phase 1) and the survival prediction model (Phase 2). In the Phase 1, the U-Net is trained with CT images and corresponding physician contours of the tumor but without survival-related information. In the Phase 2, the encoded features in the dimensional bottleneck at the middle of the U-Net are clustered by k-medoids in an unsupervised manner. The LASSO method is followed to select medoid features from the clusters based on their associations with survival. Afterward, a logistic regression model is trained for survival prediction so that survival prediction can be performed when a new patient datasets arrive with features extracted from the same U-Net. In Phase III of model validation, the model was validated for survival prediction outcome using new patients’ CT datasets.

**Figure 2:**
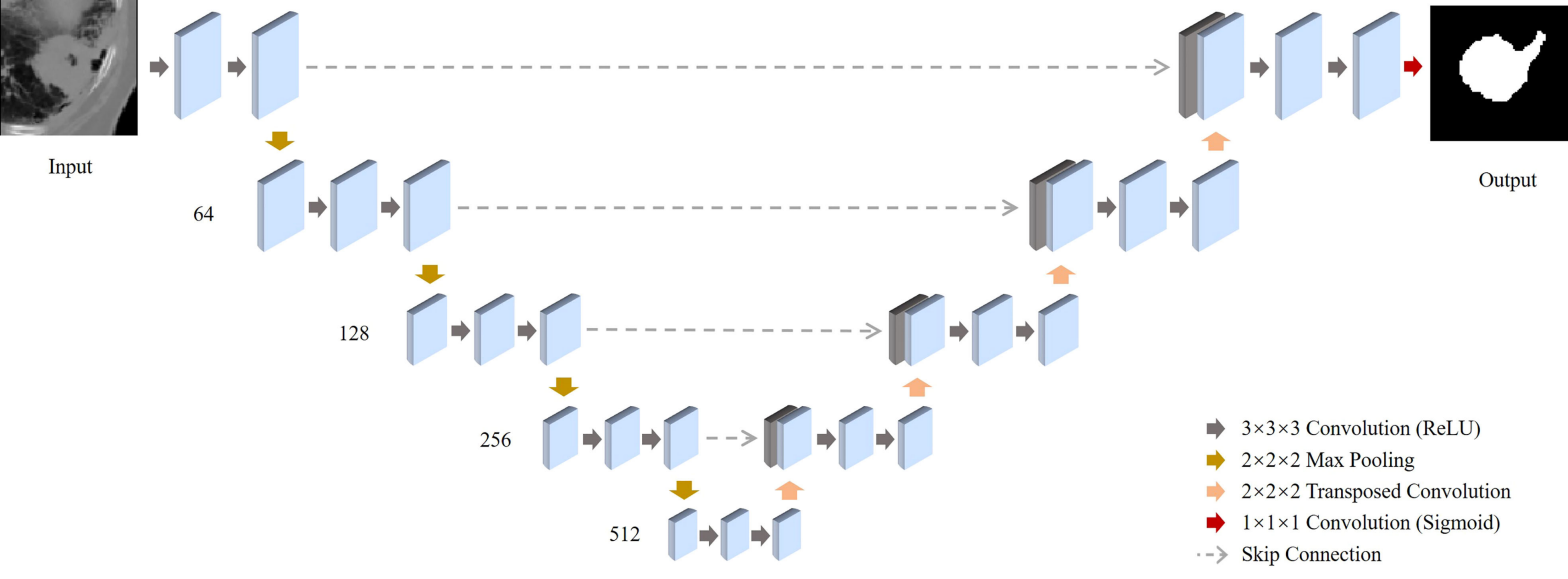
Schematic illustration of the deep-learning-based co-segmentation network with feature fusion for computed tomography (CT) co-segmentation. 3D-Unets of tumor segmentation are built for CT. All feature maps produced by all the encoders of the CT are concentrated in the corresponding decoders.

In detail, our proposed DESEP model basically is consisted of two major steps (**Figure 1**): First, a 3D CT-based tumor segmentation U-Net is developed to segment the tumor region (**Figure 2**). Second, based on the pre-trained 3D CT U-Net, we extract image features from the central latent vector, which may contain a correlation with LPFS, OS, and DSS. After feature selection by LASSO method, a total of 48 CT U-Net features and 64 PET U-Net features were retained. The 48 CT features are extracted from the CT segmentation U-Net. The 64 PET features are extracted from the PET segmentation U-Net. Both CT and PET features were selected using the same approach *via* clustering and LASSO. The remaining features were utilized for training the logistic regression to predict the binary outcomes (OS, DSS, and LPFS) in parallel. Here, for survival prediction, we perform 6-fold cross validation within our institutional dataset to predict the outcomes. In each experiment, there are 64 training cases, 16 validation cases. A set of 16 test cases are reserved before the experiment independently. Our key hypothesis is that the segmentation network which produces high-quality segmentation ensures the effective image feature extraction and encoding within the central latent vector of the U-Net. To train the 3D CT U-Net, binary cross-entropy loss is utilized as the loss function while Adam optimization algorithm is selected as the optimizer. The learning rate of the Adam optimizer was 1e^-4^, while other parameters of the default setting were used in the Python Tensorflow Library. The details of the DESEP model were previously reported (13).

Our 3D segmentation U-Net consists of the encoder network and the decoder network. In the encoder network, each input image is a 3D CT/PET image with a size of 96x96x48. In the physical coordinates, it is the same representation of 96x96x48 mm3 cubic volume in the patient body. Thus, each voxel in the input image is the equivalent of a 1x1x1 mm3 cubic in the real world. In the total of four blocks are included in the encoder network. Each block contains a 3D convolution layer, a ReLU layer, and a max-pooling layer. The four 3D convolutional layers all have kernel size 3x3x3 and produces 64, 128, 256, and 512 feature maps. The four max-pooling layers have a pooling size 2x2x2 with stride 2. Thus, in the central latent vector, it produces feature activation maps in the size of 6x6x3x512. As a symmetric structure, the decoder network includes four blocks either, and each block has a deconvolutional layer, a skip connection from the encoder network, and a convolutional layer. For the deconvolutional layers, it produces 512, 256, 128, 64 feature maps and the convolutional layer produces 256, 128, 64, 32 feature maps. Last, a 3D convolutional layer with kernel size 1x1x1 together with a softmax layer produces the final output map in the size of 96x96x48x1.

As the U-Net segments the tumor region, it also encodes a large amount of image radiomic features (which include textural and geometric information) at the “bottleneck” layer which are critical to predict a binary segmentation map. In total of 55296 features (size of 6x6x3x512) are encoded in the central latent vector of each U-Net *via* the encoder network. These encoded features contain rich information about the tumor shape and texture that may be correlated with survival, cancer progression, as well as tumor recurrence. We performed an unsupervised feature selection by applying the k-medoids clustering method to cluster the U-Net features into a reduced number of representative features (i.e. medoids of the clusters) (23). For each segmentation U-Net, a total of 55296 features are encoded into the central latent vector. According to the Silhouette method, a total of 1000 latent features from the CT U-Net and 900 latent features from the PET U-Net are medoids and selected for survival prediction. Then, we use least absolute shrinkage and selection operator to identify features exhibiting strong correlations with the survival outcomes (24). Using these DESEP features, we were able to generate predictions associated with a low or high risk for overall survival (DESEP-predicted OS), disease-specific survival (DESEP-predicted DSS), and local progression free survival (DESEP-predicted LPFS).

### 2.3 Serial measurements

RECISTv1.1 criteria were utilized to categorize treatment response on follow-up CT imaging. Measurements were taken of the target lesion along the largest tumor diameter. Progression of disease (PD) was determined based on a 20% or greater increase in the diameter relative to the smallest of previously measured diameters with a minimum absolute increase of at least 5mm. A complete response (CR) was defined as a disappearance of the target lesion. A partial response (PR) was defined as a 30% or greater decrease in target lesion summed diameters relative to its baseline pre-treatment measurement. A lesion was categorized as stable disease (SD) if it did not meet any of the previous criteria.

The deep-learning based auto-segmentation model was trained to segment the tumor volume on each follow-up CT scan. To train the 3D U-Net for our DESEP model, we selected 60 cases which 38 are used for training and 22 are reserved for testing. The binary cross-entropy loss is selected as the loss function and the Adam optimization algorithm serves as the optimizer. The batch size is 4 and the learning rate is 10^-4^ respectively. The dice similarity coefficient (DSC) (25), and average symmetric surface distance (ASSD) for the performance on the test dataset has been summarized in **Table 2**. The DSC, also known as the Sørensen–Dice index or simply Dice coefficient, is a statistical tool which measures the similarity between two sets of data^1^. In contrast, the ASSD measures the differences. The ASSD is the average of all the distances from points on the boundary of the auto-segmented region to the boundary of the ground-truth, physician’s contour, and vice versa (26). See the DSC and ASSD equations and the diagrams in the **Figure 1** of the Ref (26). From this segmentation, the total volume and largest tumor diameters were calculated. Each follow-up scan was assigned a category ranging from complete response to progression of disease based on the calculated tumor volume (auto-segmentation based volumetric RECISTv1.1) using ellipsoid volumetric thresholds and the calculated tumor diameter (computer-based unidimensional RECISTv1.1) using standard thresholds. Using this method, the patient’s final categorization was defined as the worst category received on any one follow-up CT image which were obtained 2 months after completion of SBRT then every 3 months thereafter.

**Table 2:** Summary of segmentation performance of 3D U-Net.

Metric	Mean	95% C.I.
DSC	0.78	(0.69-0.87)
ASSD	1.05	(0.45-1.65)

DSC denotes dice similarity coefficient; and ASSD denotes average symmetric surface distance.

### 2.4 Statistical analysis

All statistical analyses were performed using SPSS Statistics, Version 26.0 (IBM Corp. Armonk, NY) with a two-sided α=0.05 used to establish statistical significance. The primary endpoints utilized in this study were LPFS, DSS, and OS which were all defined from the start of SBRT. Local progression was defined as having a RECISTv1.1 categorization of progressive disease at the location of the treated target lesion as measured by the physician using the largest tumor diameter. Data for RECISTv1.1 categorization were dichotomized with a distinction drawn between progression of disease versus any other category indicating non-progressive disease. Data for survival prediction were produced as a continuous probability ranging from zero to one which was then dichotomized into a low-risk group and high-risk group based on a cut-off at a 50% predicted probability of an event within 2 years after SBRT. Here, to evaluate the predictive power of the selected features, we performed a 6-fold cross-validation on our dataset for validation. In the total of 16 cases are reserved as the test dataset. Then in each experiment, we have a training dataset with 64 cases and a validation dataset with 16 cases.

Correlation between dichotomous variables was established using Cramer’s Phi which can be interpreted similarly to a correlation coefficient with a value of one indicating a perfect agreement between two variables (27). Survival curves within RECIST v1.1 and DESEP prediction-based categories were estimated with the method of Kaplan-Meier and compared statistically with log-rank tests (28). Survival differences between categories were estimated with hazard ratios (HR) obtained from Cox regression. The concordance index was calculated using Harrell’s c-statistic. A c-statistic value of 1 represents perfect concordance between DESEP predictions and survival and a value of 0.5 a lack of concordance (29). For surviving patients, their information was censored at the date of last follow-up.

## 3 Results

The method which had the relatively higher agreement with the manually measured RECISTv1.1 was the DESEP-predicted LPFS method which extracted features associated with worse local progression (φ=0.544, p=0.001). There was a reduced agreement with RECISTv1.1 categorizations when only utilizing the auto-segmentation based volumetric RECISTv1.1 method(φ=0.227, p=0.081) or when using the computer-based unidimensional RECISTv1.1 method (φ=0.184, p=0.144).

**Figure 3:**
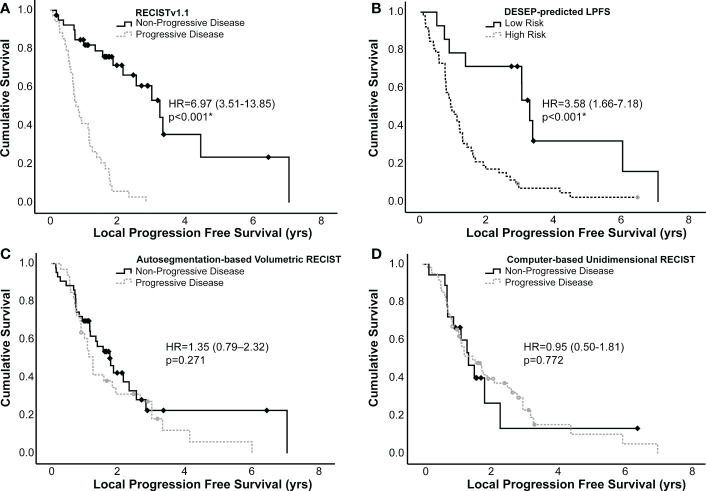
Kaplan-Meier curves generated examining local progression free survival of high and low risk groups identified using **(A)** RECISTv1.1, **(B)** DESEP-predicted LPFS, **(C)** auto-segmentation-based volumetric RECIST, and **(D)** computer-based unidimensional RECIST. RECISTv1.1, auto-segmentation-based volumetric RECIST, and computer-based unidimensional RECIST made serial measurements on multiple surveillance images to categorize patients as having progression of disease vs. non-progressive disease. DESEP-predicted LPFS extracts radiomic features associated with local progression of disease. Comparisons between groups were made using a log-rank test. An asterisk (*) denotes statistical significance.

Kaplan-Meier curves were generated to estimate differences in LPFS, these curves are presented in **Figure 3**. Having progression of disease by RECISTv1.1 was associated with worse LPFS, (HR=6.97, 3.51-13.85, c=0.70, p<0.001). Similarly, having a DESEP-predicted high risk for local progression (DESEP-predicted LPFS) was associated with a worse LPFS, (HR=3.58, 1.66-7.18, c=0.60, p<0.001). Utilizing the auto-segmentation model to simply calculate the pre-treatment tumor volume (HR=1.35, 0.79-2.32, p=0.271) or tumor diameter (HR=0.95, 0.50-1.81, p=0.772) did not show a statistically significant association with LPFS.

**Figure 4:**
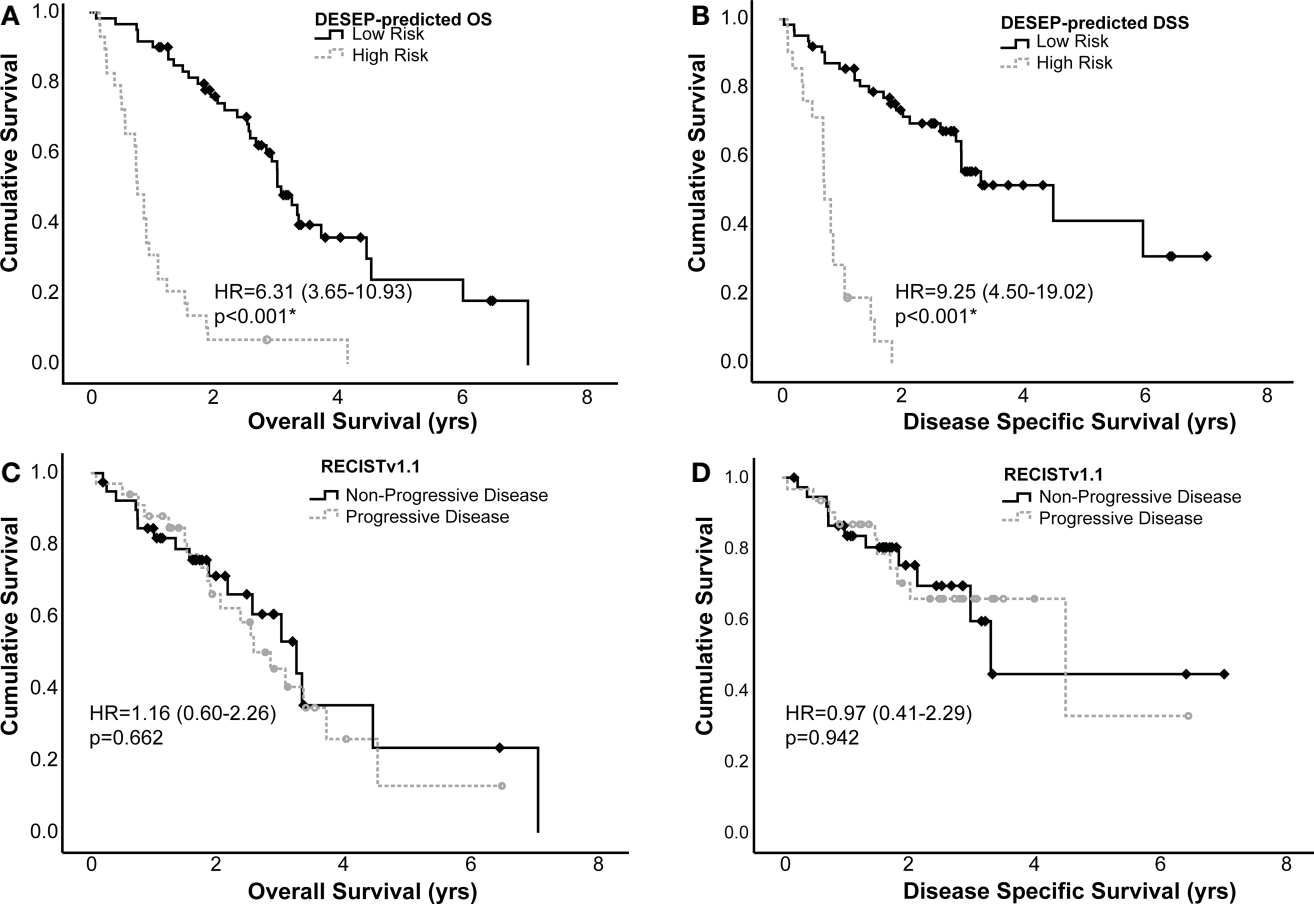
Kaplan-Meier curves examining the predictive power of DESEP-predicted categorizations for **(A)** overall survival (OS) and **(B)** disease specific survival (DSS). This is presented in comparison to RECISTv1.1 for **(C)** OS and **(D)** DSS. Both the DESEP-predicted OS and DESEP-predicted DSS methods extracted radiomic features associated with OS and DSS, respectively. RECISTv1.1 method made serial measurements on multiple surveillance images to categorize patients as having progression of disease vs. non-progressive disease, Comparisons between groups were made using a log-rank test. An asterisk (*) denotes statistical significance.

Kaplan-Meier curves were generated using both the dichotomized RECISTv1.1 and the DESEP predictions to estimate differences in OS and DSS; these curves are presented in **Figure 4**. RECISTv1.1 was unable to discriminate patients on the basis of OS (HR=1.16, 0.60-2.26, c=0.50, p=0.662) or on the basis of DSS (HR=0.97, 0.41-2.29, c=0.5, p=0.942). DESEP-predicted OS performed well when discriminating OS (HR=6.31, 3.65-10.93, c=0.71, p<0.001). **Table 3** compared mean survival time of high risk and low risk groups for three primary endpoints of OS, DSS, and LPFS. The mean OS time was 3.60 years (± 0.33 years) in the group with a predicted low risk for death compared to 1.03 years (± 0.18 years) in the high-risk group. DESEP-predicted DSS performed similarly well with DSS predictions (HR=9.25, 4.50-19.02, c=0.69, p<0.001). The mean disease specific survival time was 4.15 years (± 0.41 years) compared to 0.84 years (± 0.11 years) in the low-risk and high-risk groups respectively.

**Table 3:** Comparison of mean survival time of high risk and low risk groups for three primary endpoints of overall survival, disease specific survival, and local progression free survival.

Overall Survival					
Method	Low Risk Group (Mean ± SD)	High Risk Group (Mean ± SD)	Hazard Ratio (95%CI)	C-statistic (± SE)	p-value
DESEP-predicted OS	3.6 yrs (± 0.3 yrs)	1.0 yrs (± 0.2 yrs)	HR=6.3 (3.7-10.9)	0.71 (± 0.03)	<0.001*
RECISTv1.1	3.5 yrs (± 0.6 yrs)	3.0 yrs (± 0.4 yrs)	HR=1.2 (0.6-2.3)	0.50 (± 0.05)	0.662
Disease Specific Survival					
Method	Low Risk Group (Mean ± SD)	High Risk Group (Mean ± SD)	Hazard Ratio (95%CI)	C-statistic (± SE)	p-value
DESEP-predicted DSS	4.2 yrs (± 0.4 yrs)	0.8 yrs (± 0.1 yrs)	HR=9.3 (4.5-19.0)	0.69 (± 0.03)	<0.001*
RECISTv1.1	4.3 yrs (± 0.7 yrs)	4.0 yrs (± 0.6 yrs)	HR=1.0 (0.4-2.3)	0.50 (± 0.06)	0.942
Local Progression Free Survival					
Method	Low Risk Group (Mean ± SD)	High Risk Group (Mean ± SD)	Hazard Ratio (95%CI)	C-statistic (± SE)	p-value
DESEP-predicted LPFS	3.6 yrs (± 0.7 yrs)	1.3 yrs (± 0.2 yrs)	HR=3.6 (1.7-7.2)	0.60 (± 0.03)	<0.001*
RECISTv1.1	3.5 yrs (± 0.6ys)	1.0 yrs (± 0.1yrs)	HR=7.0 (3.5-13.9)	0.70 (± 0.03)	<0.001*

Using RECIST categorization, the high risk group was defined as having progression of disease while the low risk group was any other category indicating non-progressive disease. Comparisons between groups was performed using a log-rank test. An asterisk (*) denotes statistical significance.

**Figure 5:**
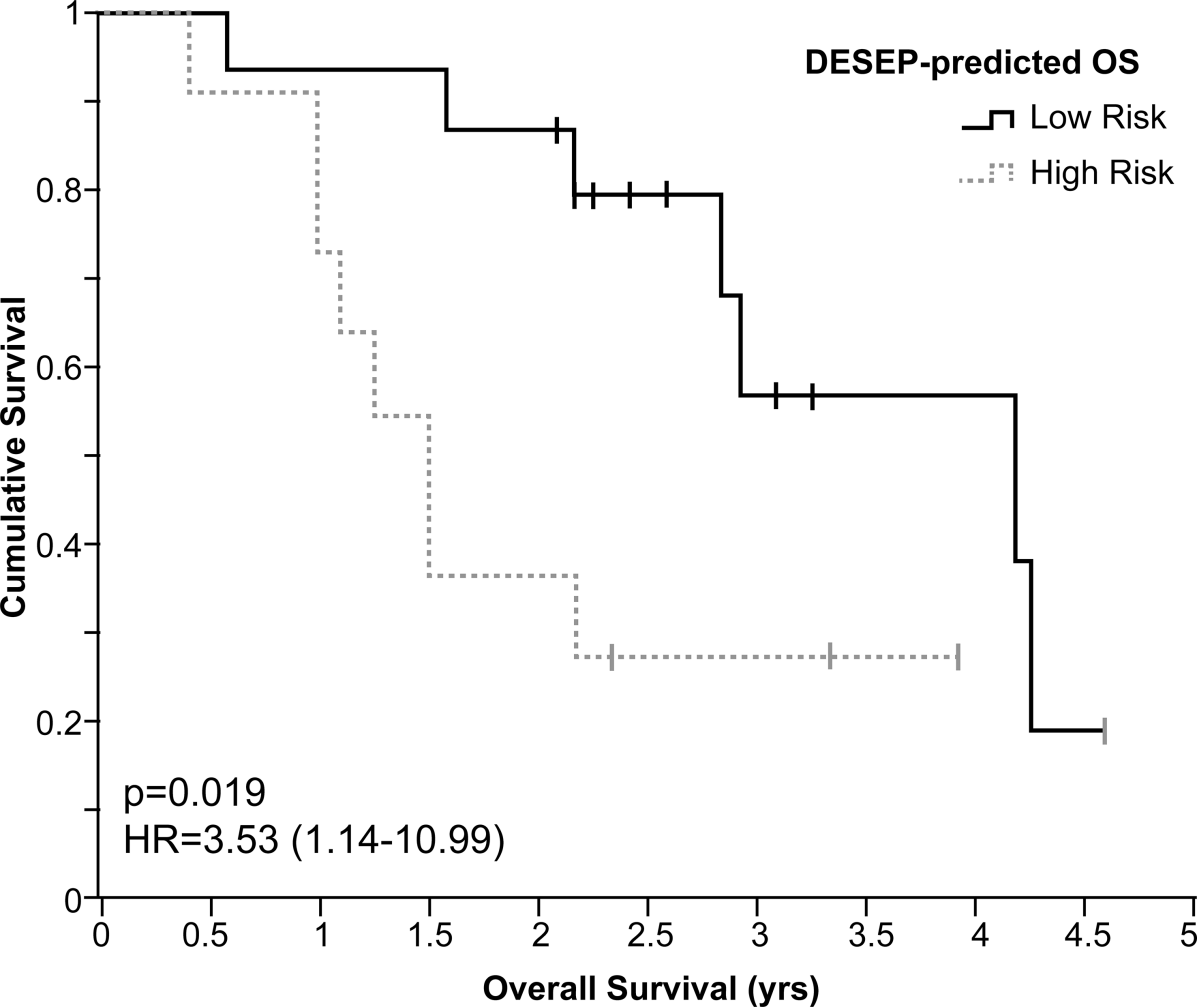
For the independent datasets of Stanford University, Kaplan-Meier curves examining the predictive power of DESEP-predicted categorizations for overall survival (OS). The low risk (solid line) represents the patients whose 2-year OS was predicted as survival, while the high risk (dash line) represents the patients whose 2-year OS was predicted as death.

We also performed validation on an external dataset provided by Stanford University. In the total of 26 NSCLC patients along with their lung CT scans and overall survival time were provided. The predictive power of the selected features from the DESEP model on the Stanford dataset is visualized in **Figure 5**. The solid line represents the predicted survival group of 2-yr OS (low risk group) by the DESEP model, while the dashed line refers to the predicted ‘dead’ group of 2-yr OS (high risk group). The hazard ratio (HR) is 3.53 with a 95% C.I. 1.14-10.99. Those two-group classified by the DESEP model showed a statistically significant difference in their Kaplan-Meyer curves (p = 0.019).

## 4 Discussion

RECISTv1.1 criteria uses linear tumor measurements identified on CT imaging to calculate simple metrics regarding the target lesion; however, medical imaging also contains a significant amount of tumor phenotypical information which can be captured with the right technological approach. Conventional radiomics include first-order features (i.e. mean, standard deviation, skewness, and kurtosis) and second-order statistical descriptors which describe textural features and statistical interrelationships between voxels (30). However, most conventional radiomic features are hand-crafted and in the form of a single metric which fail to provide sufficient information from the input image.

In patients with NSCLC, radiomic texture analysis has been shown to correlate with response to first-line chemotherapy, OS, and distant metastasis free survival (31–34). Prior work also validates the use of deep learning to extract radiomic features in CT images for tasks like pulmonary nodule detection and classification (35–38). The current study combines all of these insights into a single model which can extract information regarding tumor size, shape, texture, and volume to provide clinically relevant predictions to be used along with the RECISTv1.1 criteria to categorize treatment responses.

Multiple DESEP model-based predictions were evaluated based on their ability to provide insights regarding LPFS, DSS, and OS and compared to RECISTv1.1. In this study, RECISTv1.1 was noted to be a strong predictor of LPFS which is unsurprising since RECISTv1.1 categories are used to define local progression of disease. RECISTv1.1, however, could not discriminate patients based on their OS or DSS. The DESEP model identified patients at-risk for local progression with a high degree of accuracy and was able to categorize patients with a high- or low-risk for DSS and OS. This agrees with previous work by Xu and colleagues who noted that a deep learning model was significantly predictive of survival and cancer-specific outcomes (39). That study focused on patients with stage III NSCLC receiving concurrent chemo-radiation while the current study examined a cohort with a wide range of prognostic stages receiving SBRT. Given the more localized nature of SBRT treatment, the DESEP model input was focused only on a small bounding box centered on the target lesion in order to reduce the chance for extracting imaging characteristics which were unrelated to the treated malignancy.

The two training and validation sets are inconsistent in C. Serial Measurements and D. Statistical Analysis. The inconsistency is due to the fact that it took a few years to collect and prepare the dataset. Training of the 3D segmentation U-Nets started when only half of the dataset was prepared (n=60) while the remaining 48 cases were prepared in parallel. Thus, this study was conducted over two stages: Stage I: Training 3d U-Net (only 60 cases are prepared) and Stage II: Statistical analysis (All 108 cases are ready. In this proof-of-concept study, we used 6-fold cross-validation, instead of 10-fold cross-validation that commonly described in the literature. This was due to the lack of a sufficient number of cases. The total of 108 patients used in the study could not exactly be divided by 10 fold and thus we performed 6-fold cross-validation. For follow-up studies, the use of a larger number of subjects and 10-fold cross-validation are recommended. Textural, geometric and radiomic features were traditionally computed by experts that contain a certain amount of information from the CT images. In the previous works, we compared the performance of the DESEP model with those hand-crafted radiomic methods (13). In this study, instead of traditional hand-crafted radiomics approaches, we used deep segmentation networks to generate the features which we discovered to be correlated with the survival outcomes.

This novel deep-learning approach was most accurate when used to extract radiomic features rather than used simply to calculate the tumor volume or tumor diameter. The pioneering investigations on the feasibility of automated, quantitative tumor response assessment in neuro-oncology (RANO) using deep-learning algorithms has been previously described. Kickingereder et al. presented the promising performance of a deep learning model in automated quantitative tumor response assessment on MR images in neuro-oncology. The study was retrospectively performed through multicenter trials on 455 patients. The deep-learning model’s hazard ratio for disease progression was 2.59 compared to a hazard ratio of 2.07 for the current standard RANO. However, they did not present the comparison results in the prognostication performance for OS and DSS. Ko et al. reviewed the radiomics approaches as a possible exciting complement to RECISTv1.1 for monitoring and predicting therapeutic response (40). Studies (13, 15, 17, 20, 21, 41–47), developing hand-crafted radiomics or deep-learning models to predict clinical outcomes such as progressions, recurrences or survival rates have been conducted but they do not compare the results of the developed radiomics over the current standard RECISTv1.1 standard. To our knowledge, this is the first study to assess and compare the prediction performance of a deep-learning model and RECISTv1.1 for progression, OS, and DSS.

The literature indicates an unclear consensus regarding the use of volumetric measurement for tumor response assessment. Some prior studies indicate that volumetric assessment is more correlated with survival outcomes than RECISTv1.1 criteria, while other studies have indicated no particular benefit to volumetric assessment (11, 12, 48, 49). In the current study, using volumetric assessment did not show a benefit over RECISTv1.1 criteria at discriminating patients’ LPFS. To some extent, this can be explained by the fact that there is disagreement over the exact volumetric thresholds which should be used, and it is proposed that different disease sites may have different volumetric thresholds. Schiavon and colleagues examined multiple thresholds in their study of gastrointestinal stromal tumor including spherical thresholds (-65% & +73%) and ellipsoid thresholds (-30% & 20%) (12). Hayes and colleagues examined these same thresholds in a population of patients with NSCLC (11). In another study examining hepatic metastases, thresholds of -65% & +65% showed the best agreement with RECISTv1.1 categorization and clinical outcomes (50). An additional explanation comes from a study by Force and colleagues which noted that volumetric assessment was less beneficial than RECISTv1.1 particularly in patients with NSCLC who have received prior therapy (51). The current study uses a heterogeneous cohort of various clinical stages, and 38% had received prior chemotherapy.

There is a higher consensus in the literature regarding the reproducibility of computer-aided volumetric assessments with previous studies noting a high agreement amongst computer-aided volume segmentation (52–55). A paper by Oubel and colleagues examined a volume-based response evaluation and noticed a statically significant improvement in multi-observer agreement when compared to RECISTv1.1 criteria (56). Another major appeal of deep learning based approaches are the flexibility and rapidity of image analysis. Since the DESEP model can make prognostic predictions using imaging at only a single time point, one possible application of this model is in the analysis of surveillance imaging after radiation therapy treatment. Prior to being widely adopted in a clinical setting, CNNs contain limitations which also must be addressed. CNNs will learn a large set of patterns within images which could result in over-parameterization as only a few of these patterns are correlated with clinical outcomes. Given the data-dependent nature of the learning process of CNNs, it is paramount to maintain accurate labeling of the image data with high-quality and high-resolution. Finally, limiting the scope to only imaging data does not provide any further information about a patient’s clinical context and therefore would provide limited insights. Integrating analysis of medical imaging along with a clinical dataset can improve the model prediction performance.

The DESEP model that used in this study utilized the CT imaging datasets as sole input datasets to predict their clinical end points (OS, DSS, and LPFS), still presenting promising predictive power for this preliminary study using limited patients datasets. Even though the preliminary results were validated using the independent, external datasets of Stanford University, the robustness of predictive power is expected to suffer for large datasets obtained from different institutions. Development of the model, incorporating patients’ clinical information such as tumor-lymph node-metastasis (TNM) staging and molecular biomarkers (ALK, EGFR, PD-1) will be followed and validated for large datasets that obtained from different institutions.

## 5 Conclusion

The study provides evidence of the prognostic power of a deep-learning segmentation-based prognostication (DESEP) model in patients with NSCLC treated with SBRT for local progression-free survival (LPFS), overall survival (OS), and disease-specific survival (DSS). The progression-free survival group that was classified by the DESEP model presents a statistically significant prediction performance for LPFS (p<0.001), while it does not present a statistical correlation with OS (p=0.662) and DSS (p=0.942). In contrast to this, the DESEP model shows statistically significant predictive power for LPFS (p<0.001), OS (p<0.001), and DSS (p<0.001). The promising prognostication performance of the DESEP model was reproduced by independent, external datasets at Stanford University, classifying survival and a ‘dead’ group in their Kaplan-Meyer curves (p=0.019). The DESEP model holds the potential to be a promising complement to RECISTv1.1 criteria to determine a patient’s risk for disease progression, overall survival, and disease-specific survival. This is a proof-of-concept validation study using preliminary data. The validation of the efficacy of the deep-learning based prognostication models in a multi-institution-based prospective study using a large number of patient cases is recommended before its clinical adoption. In addition, the use of a deep learning model is recommended to be as a complement to the current clinical standard, RECISTv1.1 until its prediction robustness is fully and clinically validated.

## Data availability statement

The raw data supporting the conclusions of this article will be made available by the authors, without undue reservation.

## Author contributions

JG leads drafting of the manuscript as a first author. YH contributed to the analysis of deep-learning results, along with the development of the deep-learning algorithm. RZ, as a medical student, contributed to this study on the statistical correlation analysis on the extended data. SB significantly contributed to mentoring of deep-learning algorithm development and the interpretation of its results. XW also significantly contributed to mentoring on the development of the deep-learning algorithm, especially on the automated segmentation parts. JB contributed to this study by providing clinical expertise on RECIST and tumor response and assessment as a radiation oncologist. BA also contributed to the study design and clinical interpretation of the results as a thoracic specialty-radiation oncologist. BS contributed to the study design on statistical analysis as a biostatistician. YK initiated and led the overall study design and analysis as a corresponding author and a research project mentor of JG who is a medical resident in a radiation oncology department. All authors contributed to the article and approved the submitted version.

## Footnotes

1

$DSC=\frac{2\times \left|A\cap^{}B\right|}{\left|A\right|+\left|B\right|}$ where A denotes the ground-truth physician’s contour and B the auto-segmentation, and A $\cap^{}B$ ; denotes the intersection of A and B.
